# Impact of Primary Kidney Disease on the Effects of Empagliflozin in Patients with Chronic Kidney Disease: Secondary Analyses of the EMPA-KIDNEY trial

**DOI:** 10.1016/S2213-8587(23)00322-4

**Published:** 2023-12-04

**Authors:** Members of the EMPA-KIDNEY Collaborative Group, Executive Committee, Colin Baigent, Martin J. Landray, Christoph Wanner, William G. Herrington, Richard Haynes, Jennifer B. Green, Sibylle J. Hauske, Martina Brueckmann, Mark Hopley, Maximillian von-Eynatten, Jyothis George, Steering Committee, Executive Committee members plus National Representatives, Susanne Brenner, Alfred K. Cheung, David Preiss, Zhihong Liu, Jing Li, Laiseong Hooi, Wen Jiun Liu, Takashi Kadowaki, Masaomi Nangaku, Adeera Levin, David Cherney, Roberto Pontremoli, Aldo Pietro Maggioni, Natalie Staplin, Jonathan Emberson, Stefan Hantel, Shinya Goto, Rajat Deo, Katherine Tuttle, Parminder Judge, Kaitlin J. Mayne, Michael Hill, Sarah Y.A. Ng, Xavier Rossello, Emily Sammons, Doreen Zhu, Independent Data Monitoring Committee, Peter Sandercock, Rudolf Bilous, Charles Herzog, Paul Whelton, Janet Wittes, Derrick Bennett, Andy Burke, Richard Brown, Rejive Dayanandan, Lucy Fletcher, Hannah Gosling, Emily Harding, Richard Haynes, William G. Herrington, Parminder Judge, Carol Knott, Ryonfa Lee, Kevin Murphy, Yanru Qiao, Rachel Raff, Hui Yu, Regional Coordinating Centre Administrative Leadership and Clinical Support, YanRu Qiao, Vladimir Cejka, Marcela Fajardo-Moser, Andrea Lorimer, Donata Lucci, Anita Hepditch, Amanda Axler, Peiling Chen, Dai Hao, Cheng Beng Goh, Sarojini Sivanandam, Akiko Hashimoto, Sawako Maeno, Wakako Negoro, Aiko Tomita, Morisaki Tomoko, EMPA-KIDNEY Collaborators

**Affiliations:** Germany; United States; United Kingdom; China; Malaysia; Japan; Canada; Italy; Central Coordinating Office’s Senior Management Team based at the Clinical Trial Service Unit and Epidemiological Studies Unit, Nuffield Department of Population Health, University of Oxford; UK; Germany; Italy; US; Canada; China; Malaysia; Japan

**Keywords:** sodiumglucose co-transporter-2 inhibitor, randomised trial, cardiovascular disease, kidney function, glomerulonephritis

## Abstract

**Background:**

The EMPA-KIDNEY trial showed that empagliflozin reduced the risk of the primary composite outcome of kidney disease progression or cardiovascular death in patients with chronic kidney disease (CKD) mainly through slowing progression. We aimed to assess how effects of empagliflozin may differ by primary kidney disease across its broad population.

**Methods:**

Patients were eligible for EMPA-KIDNEY (ClinicalTrials.gov: NCT03594110) if their eGFR was 20 to <45 mL/min/1·73m^2^; or 45 to <90 mL/min/1·73m^2^ with a urinary albumin-to-creatinine ratio (uACR) of ≥200 mg/g. They were randomised 1:1 to oral empagliflozin 10mg once daily versus matching placebo. Effects on kidney disease progression (defined as a sustained ≥40% eGFR decline from randomization, end stage kidney disease, a sustained eGFR below 10 mL/min/1·73m^2^, or death from kidney failure) were assessed using pre-specified Cox models, and eGFR slope analyses used shared parameter models. Subgroup comparisons were performed by including relevant interaction terms in models.

**Findings:**

Between May 2019 and Apr 2021, 6609 participants were randomised and followed for a median of 2·0 years. Pre-specified subgroupings by primary kidney disease included 2057 (31%) with diabetic kidney disease, 1669 (25%) with glomerular disease, 1445 (22%) with hypertensive or renovascular disease, and 1438 (22%) with other or unknown causes. Overall, empagliflozin reduced the risk of kidney disease progression by 29% (empagliflozin 384/3304 *vs* placebo 504/3305; hazard ratio 0·71, 95% confidence interval [CI] 0·62-0·81), with no evidence that the relative effect size varied significantly by primary kidney disease (heterogeneity p=0.62). The between-group difference in chronic eGFR slopes (i.e. from 2 months to final follow-up) was 1·37 (95% CI 1·16 to 1·59) mL/min/1·73m^2^ per year, representing a 50% (95% CI 42-58%) reduction in the rate of chronic eGFR decline. This relative effect of empagliflozin on chronic eGFR slope, was similar in analyses by different primary kidney diseases, including in explorations by type of glomerular disease and diabetes (heterogeneity p values all >0.1).

**Interpretation:**

In a broad range patients with CKD at risk of progression, including a wide range of non-diabetic causes of CKD, empagliflozin reduced risk of kidney disease progression. Relative effect sizes were broadly similar irrespective of primary kidney disease aetiology indicating that SGLT2 inhibitors should be part of a standard of care to minimise risk of kidney failure in CKD.

**Funding:**

Boehringer Ingelheim, Eli Lilly, & MRC-UK.

## Introduction

Sodiumglucose co-transporter-2 (SGLT2) inhibitors were initially developed for the management of hyperglycaemia in people with type 2 diabetes.^1^ In chronic kidney disease (CKD), several large-scale placebo-controlled outcome trials have demonstrated that empagliflozin, dapagliflozin and canagliflozin reduced the risk of primary composite cardiorenal outcomes based on kidney disease progression or cardiovascular death.^2–4^ Meta-analysis of these and other large SGLT2 inhibitor trials demonstrated a 37% reduction in risk (relative risk [RR] 0·63, 95% confidence interval [CI] 0·58–0·69) of ≥50% sustained estimated glomerular filtration rate (eGFR) decline from randomization, end stage kidney disease (ESKD, i.e., commencement of maintenance dialysis or receipt of a kidney transplant), a sustained low eGFR (<15 or <10 mL/min per 1·73 m^2^) or death from kidney failure.^5^ Relative benefits of SGLT2 inhibition appeared similar in patients with and without diabetes despite their attenuated effect on glycosuria in the absence of hyperglycaemia.^5^

Two trials contributing to this meta-analysis included participants with non-diabetic primary kidney diseases (EMPA-KIDNEY and DAPA-CKD). Analyses from these two trials, including 476 kidney disease progression outcomes in participants with a non-diabetic cause of CKD reported that the relative effects of SGLT2 inhibition appeared similar across the different primary kidney diagnoses (P for heterogeneity between groupings of primary kidney disease=0·67).^5^ The meta-analysis did not present any details of effects of SGLT2 inhibition on eGFR slopes, albuminuria, blood pressure, hospitalisation or safety outcomes, nor specific baseline characteristics. Such details are often desired by clinicians and guideline committees to inform decisions on when to offer an SGLT2 inhibitor to particular patients.

The EMPA-KIDNEY trial assessed the effects of empagliflozin in 6609 patients with CKD, including approximately two-thirds of participants with an investigator-reported non-diabetic primary kidney disease.^2,6^ Effects among patients with different primary causes of CKD are important to consider, as different pathophysiology might be expected to respond differently to SGLT2 inhibition. We therefore aimed to assess the effects of empagliflozin on kidney outcomes among participants with different types of kidney disease using sensitive eGFR slope-based outcomes. We also provide information on the observed effects on the range of other collected outcomes.

## Methods

Details of EMPA-KIDNEY’s rationale, double-blind placebo-controlled design, protocol, pre-specified data analysis plan, completeness of follow-up and main results have been reported previously.^2,6,7^ The trial was conducted at 241 centres in 8 countries. Regulatory authorities and ethics committees for each centre approved the trial. Adults with a race-adjusted CKD-EPI^8^ eGFR of ≥20 and <45 mL/min/1·73m^2^ (irrespective of level of albuminuria); or an eGFR of ≥45 and <90 mL/min1·73m^2^ with a urinary albumin-to-creatinine ratio (uACR) ≥200 mg/g at the screening visit were eligible provided they were prescribed a clinically appropriate dose of single renin-angiotensin system (RAS) inhibitor, where indicated and tolerated. Patients with or without diabetes mellitus were eligible and polycystic kidney disease was the only excluded primary kidney disease. Those receiving at least 45 mg prednisolone daily (or equivalent) or on intravenous immunosuppression in the last 3 months were excluded.

All eligible and consenting participants entered a pre-randomization run-in phase and were provided with a 15-week supply of once daily placebo tablets. During this time, local investigators reviewed screening data, assessed current RAS inhibitor use, and approved potential participants for later randomization. Participant-reported primary kidney disease was confirmed by local lead investigators and all participants were asked if they had had a kidney biopsy. Throughout the trial, clinical responsibility for participants remained with their local doctors. After completing the run-in, willing and eligible participants had central samples of blood and urine collected for central analysis and long-term storage, and were randomly allocated to receive empagliflozin (10 mg once daily orally) or matching placebo.^9^ At follow-up visits, participants provided information on renal status (i.e. any dialysis treatment or receipt of a kidney transplant), adherence to study treatment (with reasons for stopping) and details of concomitant medication. They were also asked in a structured interview about any serious adverse events (and protocol-specified non-serious adverse events), underwent clinical measurements of blood pressure and weight, and had blood collected for safety assessments of creatinine, liver function and potassium analysed at local laboratories. Blood samples and, at selected visits, urine samples were also sent to the central laboratory for efficacy analyses (including serum creatinine) and archiving.

The pre-specified primary outcome was time-to-first occurrence of the composite outcome of kidney disease progression or cardiovascular death. Kidney disease progression included ESKD, defined as commencing maintenance dialysis or receipt of a kidney transplant; a sustained decline in eGFR to <10 mL/min1·73m^2^; a sustained decline in eGFR of ≥40% from baseline; or death from kidney failure. The term ‘sustained’ was defined as either: (i) measured at two consecutive scheduled study follow-up visits at least 30 days apart, or (ii) measured at the last scheduled study follow-up visit or the last scheduled visit before death (or withdrawal of consent or loss to follow-up). Central laboratory serum creatinine measurements were used to calculate CKD-EPI eGFR, with local laboratory creatinine measurement used when central results were missing. Hospitalisation for heart failure or death from cardiovascular causes, all-cause hospitalisations and all-cause mortality were key secondary outcomes prespecified to be adjusted for multiple testing with the use of the Hochberg step-up procedure. Kidney disease progression was a secondary outcome, and analyses of annual rate of change in eGFR (chronic and total slope) were tertiary outcomes. Further exploratory analyses of these eGFR outcomes were pre-specified. Serious acute kidney injury was based on reported adverse events and was subject to confirmation by adjudication. Pre-specified subgroups of primary kidney disease were: diabetic; glomerular; hypertensive/renovascular; other and unknown combined. To test hypotheses raised by subsidiary reports from the DAPA-CKD trial,^10,11^ the glomerular disease subgroup was further split post-hoc into IgA nephropathy (IgAN), focal segmental glomerulosclerosis (FSGS), and other glomerulonephritides (other GN).

### Statistical analyses

Follow-up was planned until at least 1070 participants had experienced a first primary outcome, in order to provide 90% power at two-sided p=0.05 to detect an 18% relative reduction in risk,^6^ but the trial was recommended to stop early at the single planned formal interim analysis for efficacy in March 2022. Results based on 990 first primary outcomes (and 888 first kidney disease progression outcomes) have been previously reported.^2^ In the present report, we focus on whether effects of SGLT2 inhibition with empagliflozin on kidney disease progression and eGFR slopes varied among participants with different types of primary kidney diseases. Secondarily, we assessed whether effects on albuminuria, blood pressure, cardiovascular, hospitalisation, and safety outcomes varied by primary disease.

All analyses were performed according to the intention-to-treat principle. Time-to-event analyses defined time at risk as originating/starting from randomization and finishing at final follow-up, or censoring at the earliest of death, loss to follow-up or withdrawal of consent. A pre-specified Cox proportional hazards regression model including adjustment for categorised baseline variables specified in the minimisation algorithm (age, sex, prior diabetes, eGFR, uACR, and geographical region) was used after testing the significance of an interaction between treatment allocation and log(survival time) to confirm no evidence against proportionality for any of the time-to-event outcomes. A treatment by primary kidney disease interaction term was then used to estimate hazard ratios (HR) and 95% CIs for empagliflozin versus placebo for time-to-event analyses (i.e., the primary, kidney disease progression, cardiovascular and safety categorical outcomes).^12^ Tests for heterogeneity between subgroups for time-to-event analyses were performed using the Wald chi-square statistic for the treatment by primary kidney disease interaction.

Effects of empagliflozin on annual rates of change in eGFR were assessed using pre-specified shared parameter models,^13^ and emphasised the chronic eGFR slope results (which take account of the reversible acute dip in eGFR when SGLT2 inhibitors are commenced) and relative effects (to enable direct tests of any differences of the effects of empagliflozin between subgroups). These relative effects on eGFR slope were calculated using methods developed for a parallel publication,^14^ and required division of the absolute effect (and its 95% CI) by the mean slope in the placebo arm. Effects of empagliflozin on continuous outcomes (i.e. blood pressure and albuminuria) used a pre-specified mixed model for repeated measures (MMRM) approach. Standard tests for heterogeneity between subgroups were performed for annual rate of change in eGFR and continuous outcomes. More complete statistical details are provided in the previously published data analysis plan and in Supplementary Materials.^2^ SAS software, version 9.4 (SAS Institute, Cary NY, USA) and R v3.6.2 were used for analyses.

### Role of funding source

The analyses were performed on the original full database developed and held by the Nuffield Department of Population Health at the University of Oxford. Boehringer Ingelheim provided a grant to the University of Oxford and have members on the Steering Committee who are responsible for reviewing all trial publications. The first and senior authors accept full responsibility for the content of the paper and the decision to submit.

## Results

### Baseline characteristics

Between May 2019 and April 2021, 6609 participants were randomised and then followed for a median of 2·0 years (IQR 1.5-2.4) years. Pre-specified subgroups of primary kidney disease included 2057 (31%) with diabetic kidney disease, 1445 (22%) with hypertensive or renovascular disease, 1669 (25%) with glomerular disease and 1438 (22%) with other or unknown causes (Table 1). Of those with glomerular disease, 817 (49%) had IgAN, 195 (12%) had FSGS and 657 (39%) had other GNs. A more detailed listing of investigator-confirmed primary kidney disease is provided in Supplementary Table 1.

Participants with glomerular disease were younger (mean age 54 [SD 14] years), and were less likely to have diabetes and cardiovascular disease than participants with other causes of kidney disease (Table 1 & Supplementary Table 1). Those with glomerular disease also had higher mean (SD) eGFR of 42 (18) mL/min/1·73m^2^ and median (IQR) uACR of 700 (306-1428) mg/g compared to the trial overall mean (SD) eGFR of 37 (14) and overall trial median (IQR) uACR of 329 (49-1069) mg/g (Table 1 & Supplementary Table 2). Among patients with glomerular disease, 1312 (79%) reported a previous kidney biopsy compared to 136 (7%), 184 (13%), and 230 (16%) of those with diabetic kidney disease, hypertensive/renovascular disease and other/unknown diagnoses, respectively (Table 1 & Supplementary Tables 1&3).

### Effects of empagliflozin by primary kidney disease

#### Kidney disease progression

**Figure 1:**
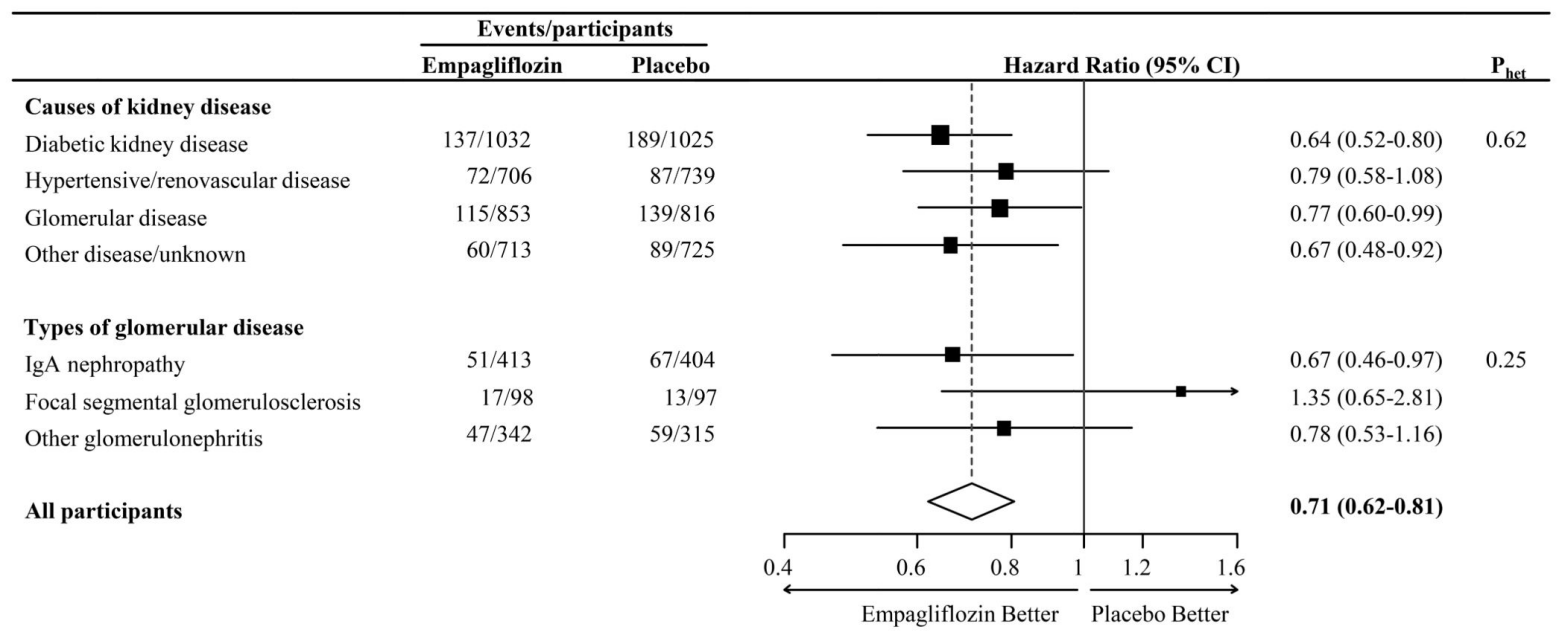
Kidney disease progression outcome by primary kidney disease A Cox proportional-hazards regression model with adjustment for baseline variables specified in the minimisation algorithm (age, sex, diabetes, estimated GFR, urinary albumin-to-creatinine ratio, and region) and a treatment by primary kidney disease interaction term was used to estimate the hazard ratios and 95% CIs for empagliflozin as compared with placebo.

Allocation to empagliflozin reduced the risk of the primary composite outcome of kidney disease progression or cardiovascular death by 28% (HR 0·72; 95% CI 0·64 to 0·82), with broadly similar effects across the four main categories of cause of kidney disease (P for heterogeneity=0·56; Table 2). Of the 990 primary outcomes, 888 participants had a kidney disease progression outcome and the 29% reduction in risk of this outcome (HR 0·71; 95% CI 0·62 to 0·81) was also similar across the kidney disease subgroups (P for heterogeneity=0·62; Table 2 and Figure 1). Further exploration found no strong evidence of heterogeneity by subtype of glomerular disease (P for heterogeneity=0·25), but were limited by only 30 outcomes in participants with FSGS (Figure 1). Risk of progression to ESKD, sustained eGFR <10 mL/min/1.73m^2^ or renal death was reduced by 31% (HR 0·69; 95% CI 0·56 to 0·85), with similar effects across the categories of kidney disease (P for heterogeneity=0.85; Table 2).

#### Annual rate of change in eGFR

**Figure 2:**
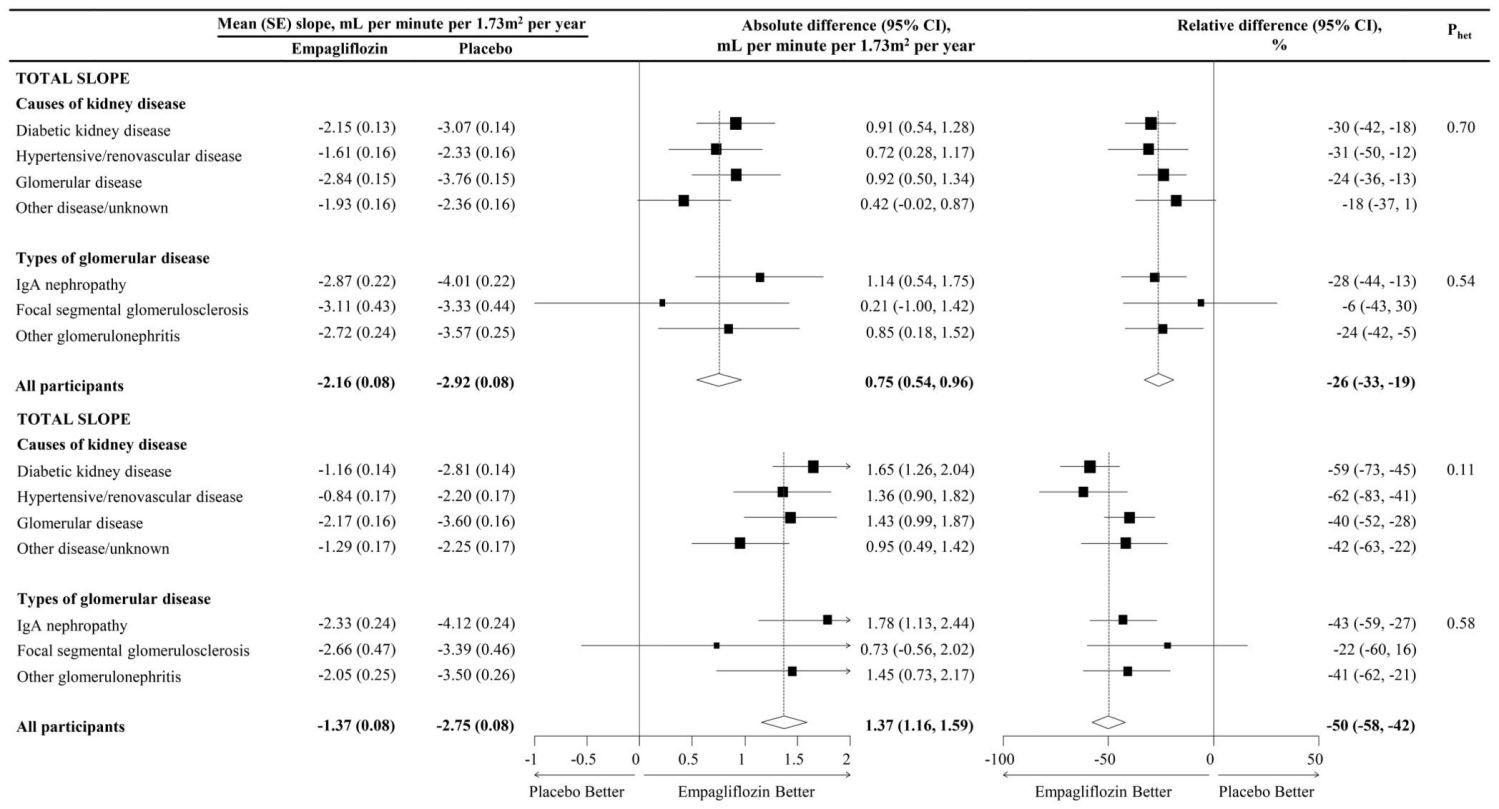
Effect of empagliflozin on annual rate of change in estimated GFR by primary kidney disease Mean annual rates of change in estimated GFR from baseline to the final follow-up visit (“total slopes”), and from 2 months to the final follow-up visit (“chronic slopes”) by treatment allocation were estimated using shared parameter models adjusted for age, sex, prior diabetes, urinary ACR category, and region. Models estimating chronic slope were additionally adjusted for baseline estimated GFR (as a continuous variable) and the interaction between baseline estimated GFR and follow-up time. This approach jointly models the annual rate of change in estimated GFR and the time to event for end-stage kidney disease (ESKD) or death. Analyses used all available central laboratory estimated GFR measurements prior to the development of ESKD. Relative difference is the absolute difference as a fraction of the mean slope in the placebo group, expressed as a percentage. The heterogeneity p values shown are calculated from the relative differences.

Analyses by annual rate of change in eGFR provides a more sensitive approach to assess for any differences in relative effects of study treatment between subgroups. The expected acute decrease in eGFR upon initiation of empagliflozin was observed (Supplementary Figure 1), followed by a slowing of the rate of annual decline. Overall, empagliflozin was associated with a slower decline in eGFR with a between-group difference in the total eGFR slope from randomization to the final follow-up visit of - 0·75 mL/min/1·73m^2^/year (95% CI, -0·54 to -0·96). When the early acute dip was excluded the between group difference in eGFR from 2 months to final follow-up (chronic-slope) was even more pronounced at -1·37 (-1·16, -1·59) mL/min/1·73m^2^/year. This effect on chronic slope represented a -50% (-42%, -58%) relative reduction in the rate of eGFR decline. This comprised -59%, -62%, -40% and -42% relative reductions in the chronic rate of eGFR decline in participants with diabetic kidney disease, hypertensive/renovascular disease, glomerular disease and other/unknown causes, respectively (P for heterogeneity across the 4 groups=0·11; Figure 2). Among those with different subtypes of glomerular disease, the relative reductions in chronic rate of eGFR decline were -43%, -22% and -41% in participants with IgAN, FSGS, and other causes of glomerular disease, respectively (P for heterogeneity across the 3 groups=0·58, Figure 2). The effects on annual rate of eGFR decline also appeared similar irrespective of diabetes type (Supplementary Figure 2), although only 68 participants had type 1 diabetes.

#### Other efficacy outcomes

Compared with placebo, empagliflozin, had no significant effect overall on a key secondary composite outcome of hospitalisation from heart failure or death from cardiovascular causes (4.0% versus 4.6%; HR 0·84; 95% CI 0·67-1·07), with similar effects regardless of primary kidney disease (P for heterogeneity=1.00; Supplementary Table 4). Overall, all-cause hospitalisations were less frequent in those allocated empagliflozin compared with placebo (24·8 vs 29·2 hospitalisations per 100 patient-years. HR 0·86; 95% CI 0·78-0·95). Effects among those with diabetic kidney disease, hypertensive/renovascular disease, glomerular disease and other/unknown causes were similar (P for heterogeneity=0.23; Supplementary Table 4). There was no significant effect on all-cause mortality between those allocated empagliflozin compared with placebo (2·28 vs 2·58 deaths per 100 patient-years. HR 0·87; 95% CI 0·70-1·08; Supplementary Table 4). Similarly, there was no evidence that empagliflozin modified risk of the tertiary outcome of major cardiovascular events overall or by primary kidney disease (P for heterogeneity=0.73; Supplementary Table 4).

#### Albuminuria and blood pressure

Geometric mean (SD) uACR was 251 (7.6), 110 (7.6), 577 (3.8), 126 (7.4) mg/g in participants with diabetic kidney disease, hypertensive/renovascular disease, glomerular disease and other/unknown causes, respectively. The difference in geometric mean (95%CI) uACR between randomized groups was -28% (-34%, -21%), -16% (-25%, -7%), -15% (-24%, -6%), and -14% (-23%, -4%) across these subgroups, respectively (Table 3), with some weak evidence that empagliflozin lowered albuminuria more in patients with diabetic kidney disease (P for heterogeneity=0.05). The overall between group differences in mean (±SE) systolic and diastolic blood pressure, were -2.6±0.3 mmHg and -0.9±0.2 mmHg, respectively (Table 3 and Supplementary Table 5). Empagliflozin appeared to have larger effects on systolic blood pressure lowering among those with diabetic kidney disease (reduction 4·1mmHg [-5·3, -2·9]; P for heterogeneity = 0.02; Table 3).

#### Safety outcomes

Overall, empagliflozin had no significant effects on risk of serious acute kidney injury (HR 0·78; 95% CI 0·60 to 1·00), with similar results across the four main categories of cause of kidney disease (P for heterogeneity=0·28; Supplementary Table 6). Ketoacidosis occurred in 6 participants allocated to empagliflozin (including five participants on insulin, one of whom had type 1 diabetes, and one participant without diabetes) and one allocated to placebo (who was not taking open-label SGLT2 inhibitor). There were 28 vs 19 lower-limb amputation events in the empagliflozin vs placebo groups, respectively (including 20 vs 14 toe amputations). These two safety outcomes mainly occurred among participants with diabetic kidney disease with too few events to provide reliable estimates of any effect in participants with non-diabetic causes of kidney disease. The incidence of serious urinary tract infection, hyperkalaemia, serious or symptomatic dehydration, severe hypoglycaemia (mainly in participants with diabetic kidney disease), liver injury and bone fractures were broadly similar between allocated treatment groups, with findings unmodified by primary kidney disease (Supplementary Table 6).

## Discussion

In these subsidiary analyses of the EMPA-KIDNEY trial, which included a large number of patients with non-diabetic causes of CKD at risk of progression, empagliflozin reduced the risk of kidney disease progression, with broadly similar sized effects in patients with different primary kidney diseases. Analyses of annual rate of change of eGFR on a relative scale enabled further detailed exploration of whether the effects of treatment vary. Such analyses suggested empagliflozin slowed decline in eGFR irrespective of these diagnoses. Empagliflozin was also generally safe and well-tolerated in the studied population.

The impact of kidney disease on the effect of SGLT2 inhibition on the progression of kidney disease observed in EMPA-KIDNEY is similar to that seen in other large placebo-controlled trials of SGLT2-inhibitors involving patients with CKD.^3–5^ A previous meta-analysis demonstrated that when standardized to the same definition of kidney disease progression, relative effect sizes are remarkably similar for a given primary cause of kidney disease. ^5^ The presented analyses add substantially to these previous data from DAPA-CKD by including information on eGFR slopes (with novel emphasis on relative effects), cardiovascular outcomes, hospitalisation, blood pressure, albuminuria, and safety in more than double the number of patients with glomerular disease, and about 3 times more participants with IgA nephropathy (with about half of participants with glomerular disease reporting Asian race).^11^ The analyses also include large numbers of patients with other non-diabetic causes of CKD, and particularly hypertensive/renovascular disease, and importantly expand the available information among patients with CKD with a uACR <200 mg/g, which were previously limited to data from the Effect of Sotagliflozin on Cardiovascular and Renal Events in Patients with Type 2 Diabetes and Moderate Renal Impairment Who Are at Cardiovascular Risk (SCORED) trial,^15^ and analyses from heart failure trials.^16–18^

The observed consistent relative effects of empagliflozin on kidney disease progression across the broad range of different primary diagnoses supports the concept of final common pathways of CKD progression. SGLT2 inhibitors restore or enhance tubuloglomerular feedback by increasing afferent arteriolar tone and improve dysregulated glomerular haemodynamics.^7^ Clinically this manifests as an acute dip in eGFR after initiation of an SGLT2 inhibitor (which is reversible on discontinuation),^6,16^ followed by a subsequent slowing in loss of eGFR longer term.^19^ Increased intraglomerular pressure and consequent glomerular hyperfiltration are hypothesised to be common to many forms of CKD,^7^ including when there is low nephron number.^7,19–22^ The presented results also may indicate possible final common pathways driven by pathophysiology in the kidney tubules. SGLT2 inhibitors decrease tubular workload and oxygen consumption via decreased reabsorption of glucose and sodium, increasing oxygen delivery capacity to the renal tubules. This may explain the larger effects of SGLT2 inhibitors on kidney disease progression than would be perhaps predicted from the more modest effects on albuminuria. This mechanism may also explain the beneficial effects on acute kidney injury observed in meta-analyses.^5,23–27^ Although there was no significant effect of empagliflozin on the risk of acute kidney injury in the EMPA-KIDNEY trial alone, the point estimate was entirely consistent with the 23% reduction in risk in meta-analyses of ~90,000 participants in 13 large SGLT2 inhibitor trials.^5^ These and other proposed mechanisms contributing to long-term kidney protection by SGLT2 inhibitors will be explored in future randomised analyses using the trial’s stored blood and urine samples and multiomic assays, and a renal MRI substudy. These explorations are important, as despite moderate-to-large reductions in risk with SGLT-2 inhibitors, residual risk remains and identifying and testing new interventions that may safely slow CKD progression remains a research priority.

Analyses of cardiovascular outcomes were limited by lower than expected numbers of events, perhaps due to recruitment of low risk populations or secular trends towards lower cardiovascular risk.^28^ This was also a feature of the DAPA-CKD trial which leaves some uncertainty about cardiovascular effects of SGLT2 inhibitors in patients with CKD without diabetes. The overall between-group difference in systolic blood pressure in EMPA-KIDNEY of -2.6 mmHg (95% CI -3.3, -1.9) was similar to the CREDENCE difference of -3.3 mmHg (95% CI -2.7, -3.9),^4^ and the DAPA-CKD difference of -2.9 mmHg (95% CI -3.6, -2,3),^36, 37^ but interestingly in EMPA-KIDNEY we observed that there may have been larger effects on systolic blood pressure in patients with diabetic kidney disease compared other primary diagnoses. Nevertheless, the proven cardiovascular benefits of SGLT2 inhibitors in heart failure populations (in which about half of the participants had decreased eGFR) did not vary by presence or absence of diabetes.^5^

Empagliflozin was generally well-tolerated and reduced the risk of hospitalisation from any cause. The risk of ketoacidosis was low and mainly among patients with diabetes on insulin at baseline. Lower limb amputations mainly occurred among participants with diabetic kidney disease. The effects on serious hyperkalaemia were not significantly different between treatment groups, but the point estimate was entirely aligned with the 17% relative reduction in the risk of hyperkalaemia from a previous meta-analysis.^29^

The results of these analyses from the EMPA-KIDNEY, DAPA-CKD and other large trials suggest widespread use of SGLT2 inhibitors should substantially reduce the future global burden of kidney failure due to both diabetic and non-diabetic primary causes of kidney disease. The consistency of findings from these and other analyses,^14^ enables simple clinical practice guidelines for patients with CKD.^30,31^ These results are particularly important for patients with CKD without diabetes who have been less well studied in the completed RAS inhibitor^32–34^ and mineralocorticoid receptor trials,^35^ and for whom serious side effects of SGLT2 inhibitors appear uncommon.^5^ SGLT2 inhibitors should become part of a standard of care for many patients with CKD.

EMPA-KIDNEY was designed to ensure findings would be widely generalisable. It also provides the largest amount of randomised data on the use of SGLT2 inhibition currently available for patients with CKD at risk of progression. A key strength of the presented analyses is the explorations using a range of statistically-sensitive approaches (i.e. eGFR slope analyses) which assessed the treatment effects on a relative scale. This approach enabled more reliable subgroup comparisons, as analyses based on absolute effects conflate any between-subgroup differences in baseline absolute risk plus any differences in the relative effect of study treatment.^14^ Limitations to the generalisability of the reported findings is the exclusion of patients with a polycystic kidney disease or a kidney transplant. Data among those with a kidney transplant will be available when the Renal Lifecycle trial reports (Clinicaltirals.gov ID: NCT05374291). Although there were particularly large numbers of participants with IgA nephropathy, there were relatively smaller numbers with other specific causes of glomerular disease, and only 68 participants with type 1 diabetes. This limited power to assess treatment effects on the range of outcomes directly in these less well-studied types of patients. The other key limitation is the relatively short median follow-up duration. Consenting EMPA-KIDNEY participants have entered a post-trial follow-up phase in which participants will be observed for the primary outcome over at least a further 2 years following completion of treatment with their randomly allocated study drug.

In summary, in a broad population of patients with CKD at risk of progression, empagliflozin safely reduced the risk of kidney disease progression and eGFR decline with broadly similar sized relative effects across patients grouped by different types of primary kidney disease.

## Writing Committee

Parminder K. Judge PhD, Natalie Staplin PhD, Kaitlin J. Mayne MBChB, Christoph Wanner MD, Jennifer B. Green MD, Sibylle J. Hauske PhD, Jonathan R. Emberson PhD, David Preiss PhD, Sarah Y.A. Ng PhD, Alistair J. Roddick MBBS, Emily Sammons MBChB, Doreen Zhu MD, Michael Hill DPhil, Will Stevens PhD, Karl Wallendszus MSc, Susanne Brenner MD, Alfred K. Cheung MD, Zhi-Hong Liu MD/PhD, Jing Li PhD, Lai Seong Hooi FRCP, Wen Jiun Liu FRCP, Takashi Kadowaki MD/PhD, Masaomi Nangaku MD/PhD, Adeera Levin MD, David Cherney MD, PhD, Aldo P. Maggioni MD, Roberto Pontremoli MD, Rajat Deo MD MTR, Shinya Goto PhD, Xavier Rossello PhD, Katherine R. Tuttle MD, Dominik Steubl MD, Dan Massey MSc, Martin J. Landray FMedSci, Colin Baigent FMedSci, Richard Haynes DM, William G. Herrington FRCP.

Drs Judge, Staplin, Haynes and Herrington contributed equally to this article, and assume responsibility for its overall content and integrity.

Affiliations of the Writing Committee are as follows: Clinical Trial Service Unit and Epidemiological Studies Unit, Nuffield Department of Population Health, University of Oxford, UK (*also part of the Medical Research Council Population Health Research Unit at the University of Oxford): PKJ, NS*, KJM, JRE*, DP*, SYAN, AJR, ES, DZ, WS, KW, MH*, MJL*, CB*, RH*, WGH*. University Clinic of Würzburg, Germany: CW & SB. Duke Clinical Research Institute, Durham, North Carolina, US: JBG. University of Utah, Salt Lake City, US: AKC. Boehringer Ingelheim International GmbH: SJH, DS. Elderbrook Solutions GmbH on behalf of Boehringer Ingelheim Pharma GmbH & Co.KG: DM. Vth Department of Medicine, University Medical Center Mannheim, University of Heidelberg, Mannheim, Germany: SJH. Department of Nephrology, Hospital Rechts der Isar, Technical University of Munich, Germany: DS. National Clinical Research Center of Kidney Diseases, Jinling Hospital, Nanjing University School of Medicine, Nanjing, China: ZHL. Fuwai Hospital, Chinese academy of Medical Sciences, National Center for Cardiovascular Diseases, Beijing, China: JL. Hospital Sultanah Aminah, Johor Bahru, Malaysia: LSH & WJL. The University of Tokyo School of Medicine/Toranomon Hospital: TK. The University of Tokyo School of Medicine, Tokyo, Japan: MN. University of British Columbia, Vancouver, Canada: AL. University of Toronto, Canada: DC. Università degli Studi and IRCCS Ospedale Policlinico San Martino di *Genova*, Italy: RP. ANMCO Research Center, Heart Care Foundation, Florence, Italy: APM. University of Pennsylvania Perelman School of Medicine, Philadelphia, US: RD. Tokai University School of Medicine, Isehara, Japan: SG. Hospital Universitario Son Espases, Health Research Institute of the Balearic Islands (IdISBa), Universitat Illes Balears (UIB), Palma de Mallorca, Islas Baleares, Spain: XR. Providence Health Care and University of Washington, US: KRT.

## Supplementary Material

Supplementary tables and Figures

## Figures and Tables

**Table 1:** Characteristics of participants at recruitment by primary kidney disease

	Diabetic kidney Disease (N= 2057)	Hypertensive/renovascular disease (N= 1445)	Glomerular disease (N= 1669)	Other/unknown (N= 1438)
**DEMOGRAPHICS**				
**Age at randomisation (years)**				
Mean (SD)	68·2 (9·8)	68·4 (11·9)	53·5 (13·6)	65·0 (14·7)
**Sex**				
Female	684 (33·3)	430 (29·8)	596 (35·7)	482 (33·5)
Male	1,373 (66.7)	1,015 (70.2)	1,073 (64.3)	956 (66.5)
**Race (all regions)**				
White	1115 (54·2)	953 (66·0)	765 (45·8)	1026(71·3)
Black	127(6·2)	85 (5·9)	22 (1·3)	28(1·9)
Asian	780 (37·9)	387 (26·8)	863 (51·7)	363 (25·2)
Mixed	7(0·3)	3 (0·2)	5 (0·3)	6 (0·4)
Other	28 (1·4)	17 (1·2)	14 (0·8)	15 (1·0)
**PRIOR DISEASE**				
**Prior diabetes**				
Yes	2057 (100·0)	402 (27·8)	172 (10·3)	409 (28·4)
No	0 (0·0)	1043 (72·2)	1497 (89·7)	1029 (71·6)
**Prior diabetes type**				
Type 1	60 (2·9)	2 (0·1)	0 (0·0)	6 (0·4)
Type 2	1977 (96·1)	397 (27·5)	168 (10·1)	394 (27·4)
Other/unknown	20 (1·0)	3 (0·2)	4(0·2)	9 (0·6)
**History of cardiovascular disease** *				
Yes	713 (34·7)	516 (35·7)	144 (8·6)	392 (27·3)
No	1344 (65·3)	929 (64·3)	1525 (91·4)	1046 (72·7)
				
**CLINICAL MEASUREMENTS**				
**Blood pressure (mmHg)**				
Mean systolic (SD)	139·9 (19·2)	138·0 (18·4)	132·8 (16·0)	134·6 (18·3)
Mean diastolic (SD)	75·2 (11·6)	78·0 (12·2)	82·0 (10·8)	77·7 (11·7)
**Body mass index (kg/m^2^)**				
Mean (SD)	31·7(7·1)	30·0 (6·3)	27·2 (5·8)	29·6 (6·7)
				
**LABORATORY MEASUREMENTS**				
**Estimated GFR (mL/min/1.73m^2^)** ^†^				
Mean (SD)	35·8 (13·9)	35·1 (11·6)	42·4(17·8)	35·7 (11·9)
<30	801 (38·9)	533 (36·9)	452 (27·1)	496 (34·5)
≥30 <45	901 (43·8)	699 (48·4)	636 (38·1)	692 (48·1)
≥45	355 (17·3)	213 (14·7)	581 (34·8)	250 (17·4)
**Urinary albumin-to-creatinine ratio (mg/g)** ^†^				
				
Geometric mean (SD)	251 (7.6)	110 (7.6)	577 (3.8)	126 (7.4)
Median (Q1-Q3)	336 (52-1304)	114 (18-623)	700 (306-1428)	149 (23-695)
<30	376 (18·3)	469 (32·5)	66 (4·0)	417 (29·0)
≥30 ≤300	623 (30·3)	444 (30·7)	344 (20·6)	453 (31·5)
>300	1058 (51·4)	532 (36·8)	1259 (75·4)	568 (39·5)
				
**CONCOMITANT MEDICATION USE**				
RAS inhibitor	1779 (86·5)	1188 (82·2)	1535 (92·0)	1126(78·3)
Immunosuppression	28 (1·4)	20 (1·4)	139 (8·3)	50 (3·5)
				
**Kidney Biopsy**	136 (6·6)	184 (12·7)	1312 (78·6)	230 (16·0)
				

Figures are n (%) or mean (SD) or median (Q1-Q3). * Defined as self-reported history of myocardial infarction, heart failure, stroke, transient ischemic attack, or peripheral arterial disease. † Uses central measurement taken at the randomization visit, or more recent local laboratory result before randomization. Prior diabetes defined as: participant-reported history of diabetes of any type, use of glucose-lowering medication or baseline HbA1c ≥48 mmol/mol at randomization visit. Abbreviations: GFR = glomerular filtration rate; RAS = renin-angiotensin system

**Table 2:** Primary and secondary outcomes by primary kidney disease

	Empagliflozin		Placebo			
	N (%)	Rate	N (%)	Rate	Hazard Ratio (95% CI)	P_het_
						
**PRIMARY OUTCOME AND ITS COMPONENTS**						
**Primary outcome: progression of kidney disease or death from cardiovascular causes**						0·56
Diabetic kidney disease	161 (15·6)	8·10	223 (21·8)	11·41	0·65 (0·53-0·80)	
Hypertensive/renovascular disease	82 (11·6)	5·95	96 (13·0)	6·92	0·82 (0·61-1·11)	
Glomerular disease	117 (13·7)	7·48	142 (17·4)	9·66	0·77 (0·60-0·98)	
Other/unknown	72 (10·1)	5·24	97(13·4)	6·84	0·73 (0·54-1·00)	
**Overall**	**432 (13·1)**	**6·85**	**558 (16·9)**	**8·96**	**0-72 (0·64**‒**0·82)**	
**Any kidney disease progression**						0·62
Diabetic kidney disease	137 (13·3)	6·89	189 (18·4)	9·67	0·64 (0·52-0·80)	
Hypertensive/renovascular disease	72 (10·2)	5·22	87 (11·8)	6·27	0·79 (0·58-1·08)	
Glomerular disease	115 (13·5)	7·36	139 (17·0)	9·46	0·77 (0·60-0·99)	
Other/unknown	60 (8·4)	4·37	89 (12·3)	6·28	0·67 (0·48-0·92)	
**Overall**	**384 (11·6)**	**6·09**	**504 (15·2)**	**8·09**	**0·71 (0·62-0·81)**	
**ESKD, sustained eGFR <10 mL/min/1.73m^2^ or death from renal causes^†^**						0·85
Diabetic kidney disease	61 (5·9)	3·00	89 (8·7)	4·44	0·64 (0·46-0·88)	
Hypertensive/renovascular disease	25 (3·5)	1·79	33 (4·5)	2·34	0·73 (0·43-1·22)	
Glomerular disease	48 (5·6)	3·03	58 (7·1)	3·86	0·78 (0·53-1·15)	
Other/unknown	24 (3·4)	1·73	41 (5·7)	2·84	0·63 (0·38-1·05)	
**Overall**	**158 (4·8)**	**2·47**	**221 (6·7)**	**3·47**	**0·69 (0·56-0·85)**	
**Sustained ≥40% decline in eGFR from randomisation^‡^**						0·49
Diabetic kidney disease	125 (12·1)	6·25	174 (17·0)	8·88	0·63 (0·50-0·79)	
Hypertensive/renovascular disease	72 (10·2)	5·21	82 (11·1)	5·88	0·86 (0·62-1·18)	
Glomerular disease	107 (12·5)	6·79	136 (16·7)	9·20	0·72 (0·56-0·92)	
Other/unknown	55 (7·7)	3·99	82 (11·3)	5·76	0·68 (0·48-0·95)	
**Overall**	**359 (10·9)**	**5·67**	**474 (14·3)**	**7·58**	**0·70 (0·61-0·81)**	
						
**SECONDARY OUTCOME**						
**ESKD or death from cardiovascular causes^†^**						0·63
Diabetic kidney disease	67 (6·5)	3·28	99 (9·7)	4·91	0·65 (0·48-0·89)	
Hypertensive/renovascular disease	25 (3·5)	1·79	37 (5·0)	2·62	0·68 (0·41-1·13)	
Glomerular disease	39 (4·6)	2·45	46 (5·6)	3·05	0·78 (0·51-1·20)	
Other/unknown	32 (4·5)	2·30	35 (4·8)	2·42	0·93 (0·58-1·51)	
**Overall**	**163 (4·9)**	**2·54**	**217 (6·6)**	**3·40**	**0·73 (0·59-0·89)**	
						

Rate = Events per 100 person-years. A Cox proportional-hazards regression model with adjustment for baseline variables specified in the minimisation algorithm (age, sex, diabetes, estimated GFR, urinary albumin-to-creatinine ratio, and region) and a treatment by primary kidney disease interaction term was used to estimate the hazard ratios and 95% CIs for empagliflozin as compared with placebo. The p value shown is the p value for heterogeneity between categories of primary kidney diagnosis.

† ESKD: End-Stage Kidney Disease, defined as start of maintenance dialysis or receipt of a kidney transplant.

‡ Sustained defined as present on two consecutive scheduled study follow-up visits or last scheduled follow-up visit prior to death or final follow-up. eGFR measurements were based on central laboratory measurements, wherever available.

**Table 3:** Urinary albumin-to-creatinine ratio and blood pressure assessments by primary kidney disease

	Diabetic kidney disease (N=2057)	Hypertensive/renovascular disease (N=1445)	Glomerular disease (N=1669)	Other/unknown (N=1438)	
					P_het_
**URINARY ALBUMIN-TO-CREATININE RATIO (uACR), mg/g**					
					
Proportional reduction in study average uACR compared to placebo	-28% (-34%, -21%)	-16% (-25%, -7%)	-15% (-24%, -6%)	-14% (-23%, -4%)	0·05
					
**BLOOD PRESSURE, mmHg**					
					
Study average change in systolic blood pressure compared to placebo	-4·1 (-5·3, -2·9)	-1·7 (-3·1, -0·2)	-2·2 (-3·6, -0·8)	-1·6 (-3·1, -0·2)	0·02
					
Study average change in diastolic blood pressure compared to placebo	-1·3 (-2·0, -0·6)	0·2 (-0·7, 1·1)	-0·3 (-1·1, 0·5)	-0·2 (-1·0, 0·7)	0·05
					

Data are study-average differences (95% CI) estimated using an adjusted pre-specified MMRM approach (see supplementary statistical methods). Analysis of effects on urinary albumin-to-creatinine ratio uses central laboratory measurements at follow-up time points 2, 18, 24 and 30 months. Analysis of effects on blood pressure uses measurements obtained at follow-up time points: 2, 6, 12, 18, 24, 30 and 36 months. Analyses required participants to have at least one follow-up measurement of the outcome variable and excluded participants with missing baseline measurements (urinary albumin-to-creatinine ratio 203/6609 [3%]; no missing baseline blood pressure measurements for analysed participants)

## Data Availability

The complete de-identified patient data set used for presented analyses will be available in due course and the application system to apply to use data will open 6 months after publication. Departmental policy details can be found here: https://www.ndph.ox.ac.uk/data-access. In adherence with the Boehringer Ingelheim Policy on Transparency and Publication of Clinical Study Data, scientific and medical researchers can request access to clinical study data after publication of the primary manuscript and secondary analyses in peer-reviewed journals and regulatory and reimbursement activities are completed, normally within 1 year after the marketing application has been granted by major Regulatory Authorities. Researchers should use the https://vivli.org/ link to request access to study data and visit https://www.mystudywindow.com/msw/datasharing for further information.

## Research in Context

### Evidence before this study

We updated a previous systematic search of the MEDLINE and Embase databases via Ovid (PROSPERO CRD42022351618) to cover the period from database inception to May 30, 2023 to identify large (i.e. at least 500 participants per arm) double-blind placebo-controlled sodium glucose co-transporter-2 (SGLT2) inhibitor trials with at least six months’ follow-up. We sought reports which described effects of SGLT2 inhibition on kidney disease progression by primary kidney disease. Other than EMPA-KIDNEY, only one previous large trial of 4304 participants, DAPA-CKD, randomised patients with proteinuric chronic kidney disease (CKD) with and without diabetes and assessed effects by primary kidney disease. Of the 1398 participants without diabetes in DAPA-CKD, only 128 patients had a primary outcome, including 109 with kidney disease progression limiting statistical power to explore effects by primary kidney disease. It raised a hypothesis that primary kidney diagnosis may not modify relative treatment effects.

### Added value of this study

EMPA-KIDNEY recruited 6609 patients with CKD including 1669 (25%) with glomerular disease (817 [12%] with IgA nephropathy), 1445 (22%) with hypertensive or renovascular disease, and 1438 (22%) with other or unknown causes. There were 562 kidney disease progression outcomes in these participants with non-diabetic causes of CKD. Overall, empagliflozin was generally well-tolerated and resulted in a 29% relative risk reduction in risk of kidney disease progression (HR 0·71; 95% CI 0·62 to 0·81). There were broadly similar relative effects across these main categories of primary kidney disease and among the different subtypes of glomerular disease. No important differences in relative effects of empagliflozin were identified in explorations using eGFR slope analyses, which provided increased sensitivity to detect any variation by different types of primary kidney disease.

### Implications of all the available evidence

Subgroup analyses from both the EMPA-KIDNEY and DAPA-CKD trials demonstrate that SGLT2 inhibitors substantially slow the progression of CKD irrespective of the primary kidney disease. SGLT2 inhibitor use should reduce the future global burden of kidney failure should it be widely prescribed to patients with CKD at risk of progression.
